# The Influence of Heredity in the Production of Crime; and the Punishment of Hereditary or Confirmed Criminals

**Published:** 1888-05

**Authors:** W. M. Yandell

**Affiliations:** El Paso


### THE INFLUENCE OF HEREDITY

*In the Production of Crime and the Punishment of Hereditary or Confirmed Criminals.*

[Prize Essays Gross Medical College.]

*By W. M. Yandell., M. D.*

Heredity, "the biological law by which living beings tend to repeat themselves in their decendants," or, "the transmission of the psychical and physical qualities of parents to their offspring," has, in its influence on crime, been studied and written of by a number of able men, but if their conclusions have been acted upon by any law making body in this country I am unaware of it.

Even a cursory knowledge of this law in relation to crime, leads irresistibly to the conclusion that our laws are radically wrong in conception in regard to the punishment of the hereditary criminal class.

It is not within the scope of this paper to more than glance at the salient points of the subject, a systematic exposition not being thought of; and I shall endeavor, as far as possible, to confine myself to heredity in connection with crime, its influence in the production of the consumptive, scrofulous, cancerous and other dyscrasiæ being eliminated, except incidentally.

The great difficulty that I encounter is, not in finding sufficient data on which to base my conclusions, but in compressing within the limits of an essay, hurriedly prepared, the interesting facts that I have gleaned from the best sources.

It will not be denied, that while "some families are characterized by such virtues as business integrity, truthfulness, temperance and frugality, others are equally marked by dishonesty, mendacity, drunkenness and prodigality, and that vicious and criminal propensities recur in some families as a rule, with some exceptions, and in varying degrees of depravity." A low standard of morality and intelligence is the rule, but to this there are numerous exceptions.

A most remarkable example of the form of heredity under consideration "has been traced through six generations, by Dr. Dugdale, in the decendants of a depraved woman named Margaret Jakes. Of seven hundred and nine individuals, the great majority consisted of murderers, thieves, prostitutes and idiots."

From Wharton and Stille I get the following: "Nothing.,' says Mr. Hill in his work on crime, "has been more clearly proved than that crime is, to a considerable extent, hereditary." He adduces numerous cases in confirmation of the fact. One of the most striking applies to the families of three brothers, containing together fifteen members. Of these no fewer than fourteen were utterers of base coin, while the fifteenth, who appeared to be an exception to his kindred, was at last detected setting fire to his own household, which he had insured for four time its value. "Suppose each of these uttering base coin to have passed only one piece a day, and to have had a career of five years duration, (which there is reason to believe is about the average,) no fewer than twenty thousand offences might have been prevented by removing the three brothers permanently from society, before they became fathers of families." The disposition to commit crime is often unquestionably an incurable form of insanity; hence, we read of persons who are all their lives criminals, and only terminate one term of imprisonment to commence another. The case of a woman is cited by Mr. Hill who continued in a career of crime for twenty-five years; and also of another woman, fifty years of age, who had already been in prison sixty-seven times. Furthermore he refers to a woman who had been in the police cells, in Edinburgh, at least one thousand times, chiefly for acts of violence."

"But it should not be forgotten, that unless there be hereditary insanity, mere hereditary tendency to crime is no more a defense to crime, than is the doctrine of hereditability of sin."

"Dr. Steinau in his essay on hereditary disease, mentions a very interesting incident, bearing on this point. "When I was a boy, there lived in my town an old man named P., who was such an inveterate thief that he went in the whole town by that name ; people speaking of him used no other appellation but that of "the thief," and everybody then knew who was meant. Children and common people were accustomed to call him by that name, even in his presence, as if they knew not his other name, and he bore it to a certain degree with much good natural forbearance. It was customary for the tradesmen and dealers who frequented the annual fair in the place, to enter into a formal treaty with him ; that is, they gave him a trifling sum of money, for which he engaged not only not to touch their property himself, but even to guard it against other thieves. A son of this P., named Charles, afterwards lived in B., during my residence there. He was respectably married and carried on a profitable trade, which supported him handsomely. Still, he could not help committing many robberies, quite without necessity, and merely from an irresistible inclination. He was several times arrested and punished ; the consequence was that he lost his credit and reputation, by which he was at last actually ruined. He died while still a young man, in the house of correction at Sp., where he had been confined for his last robbery. A son of this Charles, a grandson of the above mentioned and notorious P., in my native town, lived in the house where I resided. In his earliest youth before he was able to distinguish between good and evil, the disposition to stealing and the ingenuity of an expert thief, began already to develop themselves in him. When about three years old he stole all kind of eatables within his reach, although he had plenty to eat and needed only to ask for whatever he wanted. He therefore was unable to eat all he had taken, nevertheless he took it and distributed it among his playfellows. When playing with them some of their playthings frequently disappeared in a moment, and he contrived to conceal them for days, often for weeks, with a slyness and sagacity remarkable for his age. When about five years old he began to steal copper coins ; and at the age of six years he began to know something of the value of money and he looked out for silver pieces ; and in his eighth year he only contented himself with larger coins, and proved to be on public promenades an expert pick-pocket.

He was early apprenticed to learn a trade, but his master being continually robbed by him, soon dismissed him. This was the case with several other tradesmen, till at last, in his fourteenth year, he was committed to the house of correction."

Maudsly says, in "Body and Mind": "The observations of intelligent prison surgeons are tending more and more to prove that a considerable portion of criminals are weak-minded or epileptic or come of families in which insanity, epilepsy, or some other neurosis, exists. Mr. Thompson, surgeon to the general prison of Scotland, has gone so far recently as to express his conviction that the principal business of prison surgeons must always be with mental defects, or diseases ; that the diseases and causes of death among the prisoners are chiefly of the nervous system ; and in fine, that the treatment of crime is a branch of psychology. He holds that there is among criminals a distict and incurable *criminal class*, marked by peculiar low physical and mental characteristics ; that crime is hereditary in the families of criminals belonging to this class, and that this hereditary crime is a disorder of the mind, having close relations of nature and descent to epilepsy, dipsomania, insanity and other forms of degeneracy."

"Such criminals are really *morbid varieties* and often exhibit marks of physical degeneration—spinal deformities, cleft palate, stammering, imperfect organs of speech, hare-lip, club-foot, deafness, paralysis, epilepsy and scrofula."

Moreau relates a striking case, which is of interest as indicating the alliance between morbid or degenerate varieties and which I may quote here : "Mrs. D., aged 32 years. Her grandfather kept an inn at the time of the great French revolution, and during the Reign of Terror he had profited by the critical situation in which many nobles of the department found themselves, to get them secretly into his house, where he was believed to have robbed and murdered them. His daughter who was in his secrets, having quarreled with him, denounced him to the authorities, but he escaped conviction from the want of proofs. She subsequently committed suicide. One of her brothers had nearly murdered her with a knife on one occasion, and another brother hanged himself. Her sister was epileptic, imbecile and paroxysmally violent. Her daughter, after swimming in the head, noises in the ears, flashes before the eyes, became deranged, fancying that people were plotting against her, purchasing arms and barricading herself in her room, and was finally put in an asylum.

Thus, there were, in different members of this family, crime, melancholia, epilepsy, suicide and mania. Need we wonder at it? The moral element is an essential element of a sound and complete character; he who is destitute of it, being to that extent unquestionably a defective being, is therefore on the road to, or marks race degeneracy, and it is not a matter of much wonder that his children should, when better influences do not intervene to check the morbid tendency, exhibit a further degree of degeneracy and be actual morbid varieties. I think that no one who has studdied closely the causation of insanity will question this mode of production.

"It is an indisputable although extreme fact, that certain human beings are born with such a native deficiency of mind that all the training and education in the world will not raise them to the height of brutes; and I believe it to be not less true, that in consequence of evil ancestral influences, individuals are born with such a flaw or warp of nature that all the care in the world will not prevent them from being vicious or criminal, or becoming insane. Education, it is true, may do much, and the circumstances of life may do much, but we cannot forget that the foundation on which the acquisitions of education must rest are not acquired, but inherited. No one can escape the tyranny of his organization; no one can elude the destiny that is innate in him, and which unconsciously and irresistibly shapes his ends, even when he believes that he is determining them with consummate foresight and skill."

The foregoing interesting facts I have selected from a great number, but they are amply sufficient to base the argument on for a radical change in the treatment of incurably insane or irretrievably vicious criminals.

The paramount object in the punishment of crime being the protection of society, reformation of the criminal secondary, it follows that where there can be no reformation, the only end in view must be the protection of society.

That society is protected by the temporary confinement of professional thieves, released at the end of their terms to re-commence the commission of crimes, is not true, except for their terms of imprisonment.

If society is not protected, and the reformation of such criminals is hopeless, it follows that, except for the example in deterring others from crime, such punishment is purely vindictive—a thing not in accord with the enlightened spirit of the age.

If the proposition that the protection of society is the paramount object in the punishment of crime be correct, it matters not whether the inherited "irresistible inclination to the commission of crime quite without necessity" be a form of insanity or not.

Whether incurably insane or irretrieveably vicious, the only reasonable treatment is PERMANENT REMOVAL FROM SOCIETY, where they can neither prey upon society nor propagate their species.

Again such punishment promises well in its effect on the "transitory mania," or, as it is commonly known, the "insanity dodge" of murderers. Just why the hereditary thief should be debarred from pleading "transitory mania," unless it is that he is unable to pay for expert testimony and first-class legal advice, is not plain.

Certainly, if a man who in a fit of jealousy shoots down a poor prostitute is possessed of a "transitory frenzy," he is liable to a recurrence of the attack at any time; he is incurable, and should be confined for life.

Murder should be made odious as petty thievery is, and its punishment as certain; the certainty, not the severity of punishment being valuable as an example.

If I have clearly indicated the salient points on which I would base an argument, I have accomplished all that I hoped for in the limits of this essay.

